# Fruit consumption and physical activity in relation to all-cause and cardiovascular mortality among 70,000 Chinese adults with pre-existing vascular disease

**DOI:** 10.1371/journal.pone.0173054

**Published:** 2017-04-12

**Authors:** Xiaocao Tian, Huaidong Du, Liming Li, Derrick Bennett, Ruqin Gao, Shanpeng Li, Shaojie Wang, Yu Guo, Zheng Bian, Ling Yang, Yiping Chen, Junshi Chen, Yan Gao, Min Weng, Zengchang Pang, Baofa Jiang, Zhengming Chen, on behalf of the China Kadoorie Biobank study

**Affiliations:** 1 School of Public Health, Shandong University, Jinan, Shandong, China; 2 Qingdao Center for Disease Prevention and Control, Qingdao, Shandong, China; 3 Medical Research Council Population Health Research Unit (MRC PHRU), Nuffield Department of Population Health, University of Oxford, Oxford, United Kingdom; 4 Clinical Trial Service Unit and Epidemiological Studies Unit (CTSU), Nuffield Department of Population Health, University of Oxford, Oxford, United Kingdom; 5 Dept. of Epidemiology, School of Public Health, Peking University Health Science Center, Beijing, China; 6 Chinese Academy of Medical Sciences, Beijing, China; 7 China National Center for Food Safety Risk Assessment, Beijing, China; 8 Suzhou CDC, Suzhou, Jiangsu Province, China; 9 Meilan CDC, Haikou, Hainan Province, China; Huazhong University of Science and Technology, CHINA

## Abstract

**Objectives:**

To assess the associations of fresh fruit consumption and total physical activity with all-cause and cardiovascular mortality among Chinese adults who have been diagnosed with cardiovascular disease (CVD) or hypertension.

**Methods:**

During 2004–08, the China Kadoorie Biobank study recruited 70,047 adults, aged 30–79 years, with physician-diagnosed stroke or transient ischaemic attack, ischemic heart disease, or hypertension. Information on diet and physical activity was collected using an interviewer-administered electronic questionnaire. Cox regression was used to yield hazard ratios (HRs) for the independent and joint associations of fresh fruit consumption and total physical activity with mortality.

**Results:**

At baseline, 32.9% of participants consumed fresh fruit regularly (i.e. >3 days/week) and the mean total physical activity were 15.8 (SD = 11.8) MET-hr/day. During ~7-years follow-up, 6569 deaths occurred with 3563 from CVD. Compared to participants with <1 day/week fruit consumption, regular consumers had HR (95% CI) of 0.84 (0.79–0.89) for all-cause mortality and 0.79 (0.73–0.86) for CVD mortality. The HRs for the top vs bottom tertile of physical activity were 0.68 (0.64–0.72) and 0.65 (0.60–0.71), respectively, with no clear evidence of reverse causality. After correcting for regression dilution, each 100 g/day usual consumption of fresh fruit or 10 MET-hr/day usual levels of physical activity was associated with 23–29% lower mortality. The combination of regular fruit consumption with top 3^rd^ of physical activity (>16.53 MET-hr/day) was associated with about 40% lower mortality.

**Conclusion:**

Among Chinese adults with pre-existing vascular disease, higher physical activity and fruit consumption were both independently and jointly associated with lower mortality.

## Introduction

Despite the progressive decline in age-standardised adult mortality over the last half century, cardiovascular disease (CVD) remains a major cause of death worldwide [1]. Individuals with pre-existing CVD are at particularly increased risk of premature death. Current guidelines for secondary CVD prevention generally recommend a healthy lifestyle, particularly a diet rich in fresh fruit and vegetables and regular physical activity [2, 3]. Such recommendations, however, are mainly based on data either from studies in general population who were largely free of CVD at the start of the study [4, 5] or from relatively short-term rehabilitation trials [6–8]. There is currently insufficient high quality data showing the long-term effects of fresh fruit consumption and physical activity on mortality among individuals with pre-existing CVD, or hypertension. For practical reasons, large randomized intervention trials of lifestyle changes are difficult to conduct, particularly in low- and middle-income countries, such as China [9]. Well-performed large-scale population-based prospective cohort studies can help to assess the potential long-term health impacts of diet and physical activity among people with pre-existing vascular disease [10, 11].

In the China Kadoorie Biobank (CKB) study [12], both fresh fruit consumption [4] and total physical activity [13] have been strongly and inversely associated CVD mortality in people without CVD at baseline. The current analysis explored their relationships with all-cause and CVD mortality among people who have been previously diagnosed with CVD or hypertension. Including hypertensive CVD-free participants allowed us to compare the associations between individuals with and without manifest CVD at baseline, thus to obtain more insight on the potential effect of reverse causality (i.e. individuals with CVD may be less likely to engage in physical activity due to disease) [14].

## Materials and methods

### Study population

Details of the CKB design, survey methods, and participant characteristics have been reported previously [12]. Briefly, baseline survey was conducted in 10 geographically diverse regions (5 urban and 5 rural) in China, chosen to cover a wide range of risk exposures and disease patterns, all with good quality death and disease registries and local capacity. Between June 2004 and July 2008, all permanent residents aged 35–74 years with no severe disability were invited to participate in the study, and about one in three responded. Overall 512,891 were recruited, including a few slightly outside the targeted age range (30–34 or 75–79 years), and all provided written informed consent. Ethics approval was obtained from the Oxford University Tropical Research Ethics Committee (OXTREC), Chinese Academy of Medical Sciences Ethical Review Committee, Chinese Center for Disease Control and Prevention (China CDC) Ethical Review Committee, and the scientific review boards in each of the 10 regional centres (i.e. CDCs in Qingdao, Heilongjiang, Hainan, Jiangsu, Guangxi, Sichuan, Gansu, Henan, Zhejiang and Hunan).

Among the CKB participants, at baseline 23,129 reported having physician-diagnosed CVD (i.e. either ischaemic heart disease (IHD), stroke or transient ischaemic attack [TIA], or both) and another 48,562 participants reported having hypertension. After excluding those individuals who reported either zero physical activity (n = 1464) or being disabled (i.e. were unable to or had very limited ability to engage in physical activity, n = 180), the present analysis included 70,047 participants, of which 22,107 had CVD.

### Data collection

At local assessment clinics, trained health workers administered a laptop-based questionnaire on socio-demographic status, smoking, alcohol consumption, diet, physical activity, and personal and family medical history and measured height, weight, blood pressure etc. Dietary data covered 12 major food groups (including rice, wheat, other staple foods, red meat, poultry, fish, eggs, dairy products, fresh fruit, fresh vegetables, soybean, and preserved vegetables), with frequency of intake in 5 categories (daily, 4–6 days/week, 1–3 days/week, monthly, or never/rarely) [4]. Information about type, frequency, and duration of occupational, commuting-related, household and active recreational (leisure-time) physical activities were used to calculate total physical activity in MET hours per day (MET-hr/day) [15]. Following the completion of the baseline survey, in 2008 and 2013–14 two resurveys were undertaken among randomly selected ~5% surviving participants using similar procedures. In the second resurvey, in addition to the consumption frequency, information on the amount consumed was also collected, enabling the estimation of average consumption for each baseline level of fresh fruit category and to correct for regression dilution bias (S1 Table) [4, 16].

### Mortality follow-up

Vital status of each participant was obtained periodically through China CDC’s Disease Surveillance Points (DSP) system [17], and checked annually against local residential records, health insurance records, and by active confirmation through street committee or village administrators. In each area, the DSP system provides complete and reliable death registration, in which almost all deaths were medically certified. For the few (~5%) deaths without relevant medical attention prior to death, standardized procedures were used to determine probable causes of death from symptoms or signs described by informants (usually family members). Trained DSP staff coded all diseases on death certificates and assigned an underlying cause using ICD-10. The information entered into the CKB follow-up system (including scanned images of original death certificates) was reviewed centrally by study clinicians, blinded to baseline information [12]. For the current study, the main outcome measures were all-cause mortality and CVD mortality (ICD-10: I00-I25, I27-I88 & I95-I99). Follow-up time of each participant was calculated from the date of enrollment until death, loss to follow-up (n = 436, ~0.6%) or censoring date (31 Dec 2013).

### Statistical analysis

Multiple linear (for continuous outcomes) or logistic regression (for binary outcomes) were used to compare age, sex, and region adjusted means (standard deviations) or percentages of various baseline characteristics by levels of fresh fruit consumption and total physical activity and by type of baseline disease.

Cox regression analysis, stratified by age-at-risk (5-year intervals), sex, region (10 study areas), and baseline CVD status, was used to calculate the hazard ratios (HRs) and 95% confidence intervals (CIs) for mortality by fruit consumption or physical activity, adjusting for education, annual household income, smoking, alcohol, consumption of meat, dairy products and preserved vegetables (used as a proxy marker of salt consumption), survey season, family history of CVD, use of CVD medication, and poor health status (defined as either poor self-rated general health or usually become short of breath or have to slow down due to chest discomfort if walking on level ground). Fruit consumption and physical activity were also mutually adjusted for each other. The proportional hazard assumption was fulfilled, as similar HRs were observed in the first and second half of follow-up. In order to investigate their joint associations with mortality, participants were classified into 6 categories according to fruit consumption (> 3 days/week or not) and physical activity (in tertiles), and same as above described Cox regression models were used. In all these analyses, the floating absolute risk method was used to provide variance of log risk for each group (including the reference group) to facilitate comparisons between different exposure groups [18]. Using the fruit consumption data collected at baseline and 2 resurveys, we estimated mean usual fruit consumption for each baseline consumption group (S1 Table) and assigned these mean values to each individual participant in order to estimate the regression dilution bias-corrected HRs (95% CIs) for mortality per 1 daily portion [4, 19, 20]. The regression dilution ratio for physical activity was derived from the correlation coefficient between physical activity estimated at baseline and the first resurvey, which was 0.54. The linear associations of each 10 MET-hr/day physical activity with mortality were corrected for regression dilution bias by dividing the log HRs and 95% CIs by this regression dilution ratio.

In order to investigate the potential influence of reverse causality on the associations of fruit consumption and particularly physical activity with mortality, stratified analyses by baseline CVD status were performed. Statistical significance of effect modifications by baseline CVD status was examined through including an interaction term in the Cox regression analyses. In order to further explore the impact of reverse causality and assess the robustness of the findings, sensitivity analyses were performed by excluding the first 2 years of follow-up, participants with poor health status at baseline and those with prevalent cancer (n = 180), and participants with prevalent diabetes (either self-reported physician-diagnosed or screen-detected, n = 10,074). Moreover, additional adjustments were also done for other dietary variables (e.g. fresh vegetables and whole-grain staple foods); participants (n = 109,682) who had no self-reported prior history of hypertension or CVD at baseline but had measured SBP/DBP of >140/90 mmHg were also included; and analyses were also conducted on non-fatal CVD hospitalizations (collected through linkages with disease registries and health insurance databases [4]). All statistical analyses were performed using SAS (version 9.2), and figures were created using R version 3.0.2.

## Results

The mean (SD) baseline age was 58.9 (9.3) years, 60.3% of the participants were women, and 54.4% came from urban areas (Table 1). The mean (SD) total physical activity level was 15.8 (11.8) MET-hr/day and 32.9% consumed fresh fruit regularly (>3 days/week).

**Table 1:** Baseline characteristics of participants by fresh fruit consumption frequency and tertiles of physical activity level.

	Fresh fruit consumption (days/week)			Physical activity (MET-hr/day)			Overall (70,047)
	< 1 (n = 25,841)	1–3 (n = 21,141)	>3 (n = 23,065)	<8.91 (n = 23,692)	8.91–16.53 (n = 23,008)	>16.53 (n = 23,347)	
**Physical activity (SD), MET-hr/day**	16.0 (10.8)	15.9 (10.1)	15.6 (10.9)	6.7 (6.6)	12.8 (6.4)	28.2 (7.0)	15.8 (11.8)
**Age (SD), years**	59.6 (9.8)	58.5 (9.2)	58.4 (9.9)	63.0 (8.5)	59.9 (8.5)	53.8 (8.9)	58.9 (9.3)
**Women, %**	57.7	57.7	65.8	56.7	70.5	54.0	60.3
**Urban population, %**	29.6	53.6	82.9	60.7	59.9	42.5	54.4
**High school or above, %**	31.9	41.6	59.1	43.5	46.1	41.8	43.8
**Annual income>20,000 Yuan, %**	38.0	44.4	57.5	41.9	48.5	48.8	46.4
**Current smokers, %**	26.1	20.4	14.5	19.6	20.3	21.7	20.5
**Current drinkers, %**	13.5	11.2	9.8	9.7	12.1	13.0	11.6
Regular food consumption*, %							
Fresh fruit	-	-	100%	31.3	35.7	31.9	32.9
Fresh vegetables	97.5	99.0	99.5	98.6	99.0	98.1	98.6
Preserved vegetables	23.3	23.4	23.6	23.1	22.9	24.2	23.4
Meat	39.7	47.2	55.4	46.7	48.4	46.3	47.1
Dairy products	15.6	24.8	42.9	26.0	29.6	26.5	27.4
**BMI (SD), kg/m**^**2**^	24.8 (3.6)	25.2 (3.4)	25.4 (3.7)	25.3 (3.5)	25.2 (3.4)	24.9 (3.7)	25.1 (3.5)
**Overweight**^†^**, %**	58.1	62.7	65.7	63.4	62.6	60.0	62.0
**SBP (SD), mmHg**	151.1 (24.5)	149.3 (22.8)	146.8 (24.7)	149.2 (23.9)	148.4 (23.2)	149.8 (25.2)	149.1 (23.3)
**DBP (SD), mmHg**	84.7 (12.6)	84.3 (11.8)	83.6 (12.7)	84.4 (12.3)	84.0 (11.9)	84.3 (13.0)	84.2 (12.4)
**Uncontrolled hypertension**^‡^**, %**	68.6	66.4	62.3	65.8	65.2	66.5	65.8
**Family history of CVD, %**	27.5	28.5	31.4	28.6	29.3	29.4	29.1
**Ischemic heart disease, %**	21.1	20.9	22.6	22.8	22.1	19.7	21.5
**Stroke or TIA, %**	13.2	11.6	10.1	16.1	10.6	8.3	11.7
**CVD medication**^§^, %	73.3	75.0	76.6	77.4	75.5	71.8	74.9
**Diabetes, %**	19.2	13.1	10.2	17.0	14.5	11.6	14.4
**Poor health**^¥^**, %**	30.7	24.3	21.6	31.7	24.5	20.9	25.8

BMI: Body Mass Index; CVD: Cardiovascular Disease; DBP: Diastolic blood pressure; SBP: Systolic Blood Pressure; TIA: transient ischaemic attack.

Values are either percentage or mean (SD) and were adjusted for age, sex, and study area where appropriate.

*Regular consumption means consuming food products for at least 4 days per week.

^†^Defined as BMI ≥24 kg/m^2^;

^‡^ Defined as SBP≥140 mmHg or DBP≥90 mmHg or both.

^§^ Includes use of aspirin, statins, calcium antagonist, beta-receptor blockers, ice-inhibitors, diuretics and other unspecified medicine

^¥^ Either self-rated poor health or reported having a low capacity of walk.

Fruit consumption and physical activity were inversely related to each other. Participants with higher fruit consumption were more likely to be younger, women and urban residents, had higher education and income, and less likely to be current smokers and alcohol drinkers. In contrast, participants with higher levels of physical activity were more likely to be rural residents, current smokers, and current drinkers. Comparing to participants with <1 day/week fruit consumption, regular consumers had 0.6 kg/m^2^ higher body mass index (BMI), 4.3 mmHg lower systolic blood pressure (SBP), and 1.1 mmHg lower diastolic blood pressure (DBP). Physical activity was inversely correlated with BMI (0.4 kg/m^2^ lower in the top vs bottom tertile) but had no clear association with blood pressure. Participants with stroke or TIA were more likely to be men, have lower education, income, fruit consumption and physical activity, and higher prevalence of diabetes and poor health status (S2 Table).

**Fig 1:**
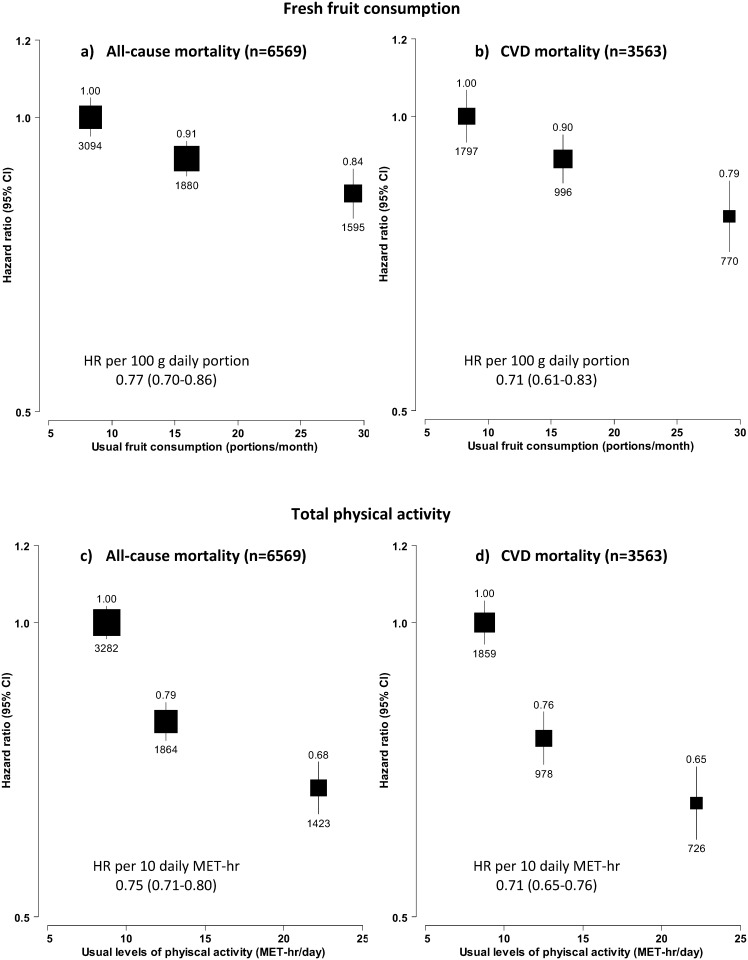
Adjusted HRs for all-cause and CVD mortality by usual levels of fresh fruit consumption and physical activity. a) and b) are the associations of fresh fruit consumption with all-cause and CVD mortality, and c) and d) are the associations of physical activity with all-cause and CVD mortality. Analyses were stratified by age-at-risk, sex, region, and baseline CVD status, and adjusted for education, income, smoking, consumption of alcohol, dairy products, meat and preserved vegetables, survey season, diabetes status, family history of CVD, CVD medication, poor health status, and fruit consumption or physical activity, where appropriate. The boxes represent hazard ratios, with the size inversely proportional to the variance of the logarithm of the hazard ratio, and the vertical lines represent 95% confidence intervals. The numbers above the vertical lines are point estimates for hazard ratios, and the numbers below the lines are numbers of events. The x-axis location of each box corresponds to the group average of usual fruit consumption or usual physical activity for each category of participants.

During the ~0.5 million person-years follow-up, 6569 participants died between age of 35–79 years, mainly from CVD (~54%), cancer (~24%) and respiratory disease (~ 7%) (S3 Table). Both fruit consumption and physical activity were significantly and inversely associated with all-cause mortality and CVD mortality (Fig 1). Overall, regular fruit consumption was associated with 16% (HR 0.84, 0.79–0.89) lower risk of all-cause mortality and 21% lower CVD mortality (HR 0.79, 0.73–0.86), with 1 daily portion (100 grams) usual consumption associated with HR of 0.77 (0.70–0.86) and 0.71 (0.61–0.83), respectively. For physical activity, the top tertile associated with 32% lower all-cause mortality (HR 0.68, 0.64–0.72) and 35% lower CVD mortality (HR 0.65, 0.60–0.71), as compared with the bottom tertile. Each 10 MET-hrs/day of usual physical activity was associated with HRs of 0.75 (0.71–0.80) and 0.71 (0.65–0.76), respectively.

**Fig 2:**
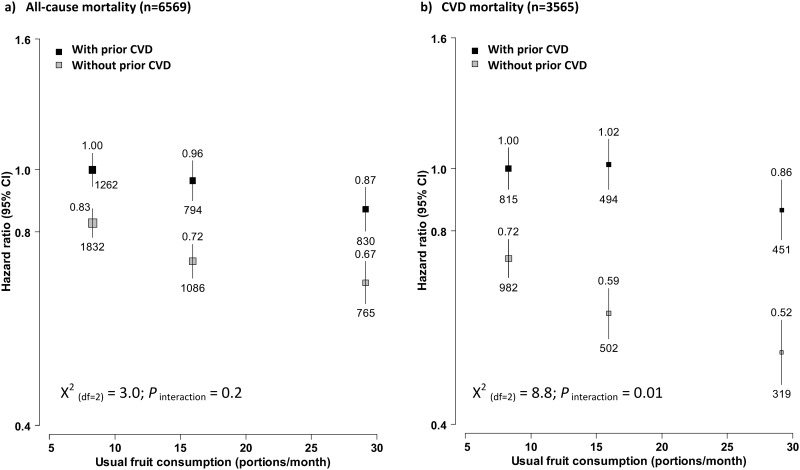
Adjusted HRs for all-cause and CVD mortality by fresh fruit consumption, stratified by baseline CVD status. Analyses were stratified by age-at-risk, sex and region, and adjusted for education, income, smoking, consumption of alcohol, dairy products, meat and preserved vegetables, survey season, diabetes status, family history of CVD, CVD medication, poor health status, and physical activity. Convention as in Fig 1. Black boxes were for participants with baseline prevalent CVD and the grey boxes were for those without prevalent CVD at baseline.

**Fig 3:**
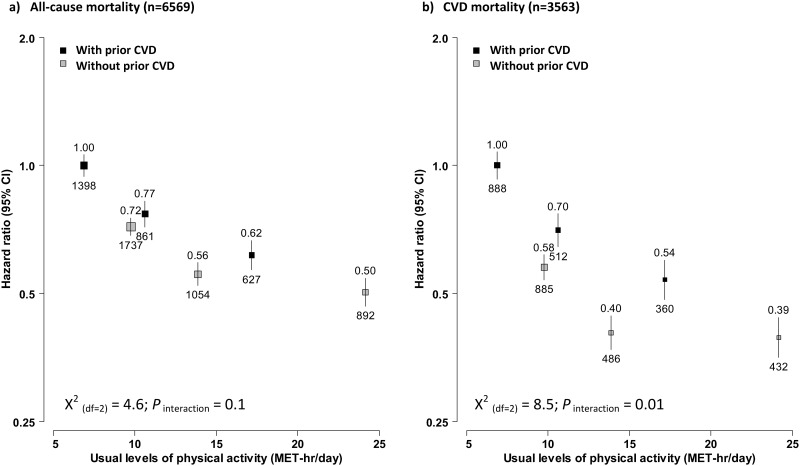
Adjusted HRs for all-cause and CVD mortality by total physical activity, stratified by baseline CVD status. Analyses were stratified by age-at-risk, sex and region, and adjusted for education, income, smoking, consumption of alcohol, dairy products, meat and preserved vegetables, survey season, diabetes status, family history of CVD, CVD medication, poor health status, and fruit consumption. Convention as in Fig 1. Black boxes were for participants with baseline prevalent CVD and the grey boxes were for those without prevalent CVD at baseline.

In stratified analyses, fruit consumption showed a similar association with mortality in participants with and without baseline prevalent CVD (Fig 2a & 2b). Compared to participants who consumed fruit <1 day/week, regular consumers had 13% lower all-cause mortality (HR = 0.87, 95% CI: 0.80–0.94) and 14% lower CVD mortality (HR 0.86, 0.77–0.96) in those with baseline CVD, whereas the corresponding HR differences among those without baseline CVD were slightly larger, as 16% (HR 0.83 vs 0.67) and 20% (0.72 vs. 0.52), respectively. For physical activity, its associations with mortality were stronger in participants with baseline CVD, with the top third having 38% lower all-cause mortality (HR 0.62, 0.57–0.67) and 46% lower CVD mortality (HR 0.54, 0.48–0.60). The corresponding HR differences in those without baseline CVD were only 22% (HR 0.50 vs. 0.72) and 19% (HR 0.39 vs. 0.58), respectively. However, the regression lines of usual physical activity with mortality for these two participant groups converged in a log-linear manner (Fig 3a & 3b).

**Fig 4:**
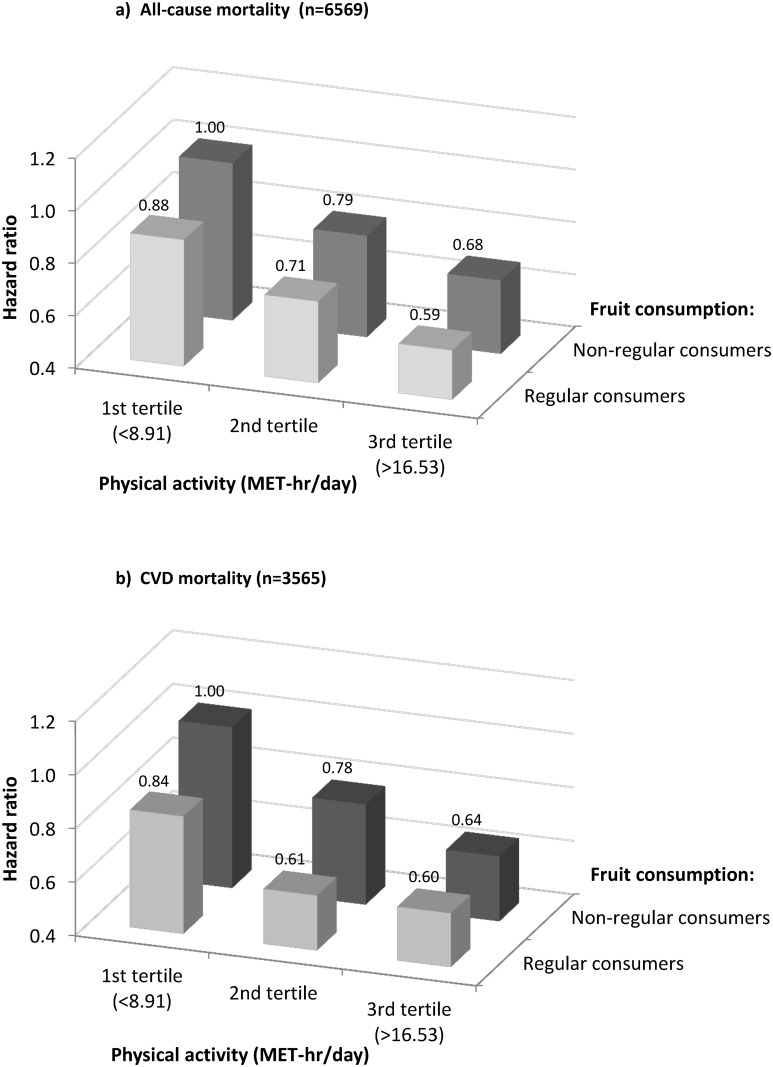
Joint associations of fruit consumption and physical activity with all-cause and CVD mortality. Analyses were stratified by age-at-risk, sex, region, and baseline CVD status, and adjusted for education, income, smoking, consumption of alcohol, dairy products, meat and preserved vegetables, survey season, diabetes status, family history of CVD, CVD medication, and poor health status.

Fig 4 shows the joint associations of fruit consumption and physical activity with mortality. Higher fruit consumption was associated with 7–12% lower risk of all-cause mortality and 4–17% lower risk of CVD mortality at each level of physical activity. For both all-cause and CVD mortality, the HR differences between regular and non-regular fruit consumers seemed slightly larger among people with lower physical activity. Compared to the least healthy group which included those participants in the lowest tertile of physical activity and also did not consume fruit regularly, any increase in fruit consumption or physical activity was associated with somewhat lower risk of mortality; and the risk was 41% (HR 0.59, 0.52–0.67) lower for all-cause mortality and 40% (HR = 0.60, 0.50–0.72) lower for CVD mortality in the most healthy group, i.e. those regular fruit consumers who also had the highest levels of physical activity. The differences in usual levels of physical activity and fruit consumption in these two extreme groups were approximately 11 (9 vs. 20) MET-hr/day and 60 grams per day respectively.

Interestingly, the association of fruit consumption with mortality tended to become stronger with increased level of SBP but the converse was found for the association of physical activity with mortality, which became weaker, although remained statistically significant (S1 and S2 Figs). The association between fruit consumption and mortality tended to be stronger in rural than urban areas, but the associations of physical activity were largely consistent among subgroups. As shown in the S4 Table, the HRs differed little between people with IHD and stroke or TIA at baseline, although latter group had much lower levels of both fruit consumption and physical activity. None of the sensitivity analyses materially altered the observed associations (S5 Table). The associations with non-fatal CVD hospitalization were also concordant with the results from main analyses on mortality (S6 Table).

## Discussion

In this prospective investigation of over 70,000 Chinese adults with prior history of CVD or hypertension, both fresh fruit consumption and total physical activity were associated with lower all-cause and CVD mortality. These associations were broadly consistent across various subgroups of participants. Moreover, the observed inverse associations did not appear to be due to reverse causality. Jointly the combination of 60 gram/day more usual fruit consumption and 11 MET-hrs/day higher usual physical activity was associated with 40% lower mortality.

In the context of primary prevention, both fresh fruit consumption and physical activity have been associated with lower risk of CVD incidence and mortality in our [4, 13] and other mainly Western studies [21–24]. Very few observational data, however, have demonstrated such associations in people with prevalent vascular disease such as CVD [11, 25–28] and hypertension [29]. For example, in a secondary analysis of data from a trial involving more than 11,000 Italians with myocardial infarction, more than 1 day per week of fruit consumption was associated with 27% (2–46%) lower risk of all-cause mortality as compared to those who never or almost never consumed fruit [25]. This association would close to the 16% difference observed in our study if the lowest two consumption groups were combined together, given the never/almost never consumption group included only 55 out of 1658 deaths. In the EPIC-elderly study of 2671 participants with myocardial infarction, consumption of fruits and nuts was significantly and inversely associated with mortality, with each 180 g/day associated with 12% lower mortality [28]. Within the CKB, higher fresh fruit consumption has been associated with lower mortality among people with prevalent diabetes [30]. Consistent findings in the current study (even after excluding participants who also had diabetes at baseline, S5 Table) reinforce the potential health benefit of fresh fruit consumption in people with cardiometabolic diseases.

For physical activity, most of the previous studies have tended to focus on leisure time exercise rather than total physical activity that also includes activities related to work, commuting and household chores. For instance, in a US cohort study of ~4000 IHD participants, participating in physical exercise at least 4 times per week was associated with 29% (14–41%) lower risk of mortality as compared to those did not exercise [11]. To the best of our knowledge, no previous study has examined the long-term health effects of total physical activity among people living with vascular disease. Compared to people in high-income countries, people in low- and middle-income countries, such as Chinese, participate in much less leisure time exercise, with occupational and household activity accounting for a much larger proportion of total physical activity [31]. Although numerous rehabilitation trials have confirmed the benefits of structured aerobic exercise in people at post-acute stage of CVD [8, 32, 33], there is a lack of data on unstructured or other types of physical activity. The dose-response relationship between total physical activity and mortality observed in the present study accords with the consensus that greater health benefits could be achieved by increasing physical activity among people who are physically less fit [34, 35]. In other words, the steeper inverse associations in participants with baseline CVD, as compared to the associations in those participants with hypertension only (CVD-free) at baseline, should be attributed to their relatively lower level of usual physical activity, rather than reverse causality.

Including a group of participants with hypertension but not manifest CVD was a unique strength of the current study, that afforded us the opportunity to investigate the potential influence of reverse causality [36]. Few previous publications investigated this important issue. Other major strengths of our current study include a larger number of community-dwelling patients who have been diagnosed with vascular diseases; high completeness of follow-up; detailed information on general health status at baseline, allowing us to perform detailed adjustment and sensitivity analyses to further explore the potential impacts of reverse causality; repeated measures on exposures during follow-up in a random sample of surviving participants enabling us to correct for regression dilution bias [37, 38].

This study also has several limitations. First, the information on fruit consumption and physical activity was collected using a general questionnaire, which has not been validated against objective measures. However, our previous work has provided some indirect evidence of validity [4, 15, 31]. Second, baseline prevalent disease status was self-reported and we have no further information to confirm/refute or sub-classify these diseases. However, a high specificity of such self-reported CVD and hypertension status could be expected [39–41]. Third, there may be some selection bias because our baseline survey did not include people who were unable to attend the assessment clinics (e.g. due to severe health conditions caused by CVD). Fourth, although we have attempted to deal with all potential confounders in our analyses, our results may still be subject to residual confounding from unknown and unmeasured factors.

In summary, our findings concur with previous data from mainly general populations with regard to the potential benefits of fresh fruit consumption and physical activity in preventing overall and cardiovascular death [42]. As the population ages, the prevalence of vascular disease will greatly increase in China and elsewhere. Although these high risk individuals may have received health education messages encouraging lifestyle changes, the prevalence of unhealthy behaviours such as smoking, alcohol, overweight, and uncontrolled hypertension are still high, as seen in the current study and previously results [43]. This poses a major challenge to public health professionals as well as clinicians and health-care systems. In addition to pharmacological therapy, guidelines to this high risk population should also integrate advice on diet and physical activity, while at the same time pay attention to other key CVD risk factors, such as smoking, diabetes and uncontrolled hypertension.

## Supporting information

S1 Fig Adjusted HRs of 1 daily portion of fresh fruit consumption associated with all-cause and CVD mortality by subgroups of participants. Analyses were stratified by age-at-risk, sex, region, and baseline CVD status, and adjusted for education, income, smoking, consumption of alcohol, dairy products, meat and preserved vegetables, survey season, diabetes status, family history of CVD, CVD medication, poor health status, and physical activity, where appropriate. The black boxes represent HRs and the horizontal bars represent their confidence intervals. The open diamonds represent the overall estimates of HRs and their confidence intervals. 1. the HR after correcting for regression dilution bias; 2. the HR before correcting for regression dilution bias.
(DOCX)

S2 Fig Adjusted HRs of 10 MET-hr/day physical activity associated with all-cause and CVD mortality by subgroups of participants. Analyses were stratified by age-at-risk, sex, region, and baseline CVD status, and adjusted for education, income, smoking, consumption of alcohol, dairy products, meat and preserved vegetables, survey season, diabetes status, family history of CVD, CVD medication, poor health status, and fruit consumption, where appropriate. The boxes represent hazard ratios and the horizontal bars represent their confidence intervals. The open diamonds represent the overall estimates of HRs and their confidence intervals. 1. the HR after correcting for regression dilution bias; 2. the HR before correcting for regression dilution bias.
(DOCX)

S1 TableCalculation of usual fruit consumption using data from the 1^st^ and the 2^nd^ resurvey (n = 2690).*The mean daily portion number came from the 2nd resurvey data, used as a proxy of baseline mean daily portion. †Usual intake amount for each group was estimated by taking into account changes in consumption frequency between baseline and 1st resurvey using this formula $\text{Un}=\sum_{\text{i}=1}^{3}\left(\text{Fni}\times \text{Bi}\right)$; F is the percentage in each cell, B is the baseline proportion per month for each baseline category, U is the usual proportion per month for each baseline category. This method for correcting for regression dilution bias is equivalent to the MacMahon-Peto method, described in detail by Frost C and Thompson SG. In ***Correcting for regression dilution bias*: *comparison of methods for a single predictor variable***.[Journal of the Royal Statistical Society Series A (Statistics in Society) 2000, 163(2):173–189].(DOCX)

S2 Table Baseline characteristics of participants by baseline prevalent disease*. Values are either percentage or mean (SD) and were adjusted for age, sex, and study area where appropriate. * Stroke group included all participants with self-reported physician-diagnosed stroke, among which 1142 also had IHD; IHD group included those with self-reported IHD, but not stroke; Hypertension group included participants with self-reported hypertension, but without stroke or IHD. † In men, the proportion of current smokers was 47.9% and the proportion of current drinkers was 27.0%; the corresponding proportions in women were 2.5% and 1.5% respectively. ‡ Regular consumption means consuming food products for at least 4 days per week. ¶ Overweight was defined as BMI≥24 kg/m^2^ and uncontrolled hypertension was defined as SBP≥140 mmHg or DBP≥90 mmHg or both. § Includes aspirin, statins, calcium antagonist, beta-receptor blockers, ice-inhibitors, diuretics or other unspecified drugs. ¥ Either self-rated poor health or reported having a low capacity of walk.
(DOCX)

S3 Table Distribution of total deaths at 35–79 years during follow-up. (DOCX)

S4 Table Separate associations of fresh fruit consumption and physical activity with all-cause and CVD mortality in people with prevalent stroke and those with IHD at baseline. IHD: Ischemic heart disease; TIA: transient ischaemic attack. *1142 participants also had IHD Analyses were stratified by age-at-risk, sex, region, and baseline CVD status, and adjusted for education, income, smoking, consumption of alcohol, dairy products, meat and preserved vegetables, survey season, diabetes status, family history of CVD, CVD medication, poor health status, and fruit consumption or physical activity, where appropriate.
(DOCX)

S5 Table Results from sensitivity analyses. Analyses were stratified by age-at-risk, sex, region, and baseline CVD status, and adjusted for education, income, smoking, consumption of alcohol, dairy products, meat and preserved vegetables, survey season, diabetes status, family history of CVD, CVD medication, poor health status, and fruit consumption or physical activity, where appropriate.
(DOCX)

S6 Table Fresh fruit consumption and physical activity in relation to non-fatal CVD events. Analyses were stratified by age-at-risk, sex, region, and baseline CVD status, and adjusted for education, income, smoking, consumption of alcohol, dairy products, meat and preserved vegetables, survey season, diabetes status, family history of CVD, CVD medication, poor health status, and fruit consumption or physical activity, where appropriate.
(DOCX)

## References

[1] Lozano R, Naghavi M, Foreman K, Lim S, Shibuya K, Aboyans V, et al Global and regional mortality from 235 causes of death for 20 age groups in 1990 and 2010: a systematic analysis for the Global Burden of Disease Study 2010. Lancet. 2012;380(9859):2095–128. Epub 2012/12/19. 10.1016/S0140-6736(12)61728-0 23245604 PMC10790329

[2] Jones K, Saxon L, Cunningham W, Adams P. Secondary prevention for patients after a myocardial infarction: summary of updated NICE guidance. BMJ. 2013;347:f6544 Epub 2013/11/15. 10.1136/bmj.f6544 24227827

[3] Lawrence M, Fraser H, Woods C, McCall J. Secondary prevention of stroke and transient ischaemic attack. Nurs Stand. 2011;26(9):41–6. Epub 2011/12/15. 10.7748/ns2011.11.26.9.41.c8800 22165550

[4] Du H, Li L, Bennett D, Guo Y, Key TJ, Bian Z, et al Fresh Fruit Consumption and Major Cardiovascular Disease in China. N Engl J Med. 2016;374(14):1332–43. Epub 2016/04/07. 10.1056/NEJMoa1501451 27050205 PMC4896382

[5] Li J, Siegrist J. Physical activity and risk of cardiovascular disease--a meta-analysis of prospective cohort studies. Int J Environ Res Public Health. 2012;9(2):391–407. Epub 2012/04/04. 10.3390/ijerph9020391 22470299 PMC3315253

[6] Greenlund KJ, Giles WH, Keenan NL, Croft JB, Mensah GA. Physician advice, patient actions, and health-related quality of life in secondary prevention of stroke through diet and exercise. Stroke. 2002;33(2):565–70. Epub 2002/02/02. 11823671 10.1161/hs0202.102882

[7] Heran BS, Chen JM, Ebrahim S, Moxham T, Oldridge N, Rees K, et al Exercise-based cardiac rehabilitation for coronary heart disease. Cochrane Database Syst Rev. 2011;(7):CD001800 Epub 2011/07/08. 10.1002/14651858.CD001800.pub2 21735386 PMC4229995

[8] Anderson L, Oldridge N, Thompson DR, Zwisler AD, Rees K, Martin N, et al Exercise-Based Cardiac Rehabilitation for Coronary Heart Disease: Cochrane Systematic Review and Meta-Analysis. J Am Coll Cardiol. 2016;67(1):1–12. Epub 2016/01/15. 10.1016/j.jacc.2015.10.044 26764059

[9] Gersh BJ, Sliwa K, Mayosi BM, Yusuf S. Novel therapeutic concepts: the epidemic of cardiovascular disease in the developing world: global implications. Eur Heart J. 2010;31(6):642–8. Epub 2010/02/24. 10.1093/eurheartj/ehq030 20176800

[10] Dehghan M, Mente A, Teo KK, Gao P, Sleight P, Dagenais G, et al Relationship between healthy diet and risk of cardiovascular disease among patients on drug therapies for secondary prevention: a prospective cohort study of 31 546 high-risk individuals from 40 countries. Circulation. 2012;126(23):2705–12. Epub 2012/12/06. 10.1161/CIRCULATIONAHA.112.103234 23212996

[11] Booth JN 3rd, Levitan EB, Brown TM, Farkouh ME, Safford MM, Muntner P. Effect of sustaining lifestyle modifications (nonsmoking, weight reduction, physical activity, and mediterranean diet) after healing of myocardial infarction, percutaneous intervention, or coronary bypass (from the REasons for Geographic and Racial Differences in Stroke Study). Am J Cardiol. 2014;113(12):1933–40. Epub 2014/05/06. 10.1016/j.amjcard.2014.03.033 24793668 PMC4348576

[12] Chen Z, Chen J, Collins R, Guo Y, Peto R, Wu F, et al China Kadoorie Biobank of 0.5 million people: survey methods, baseline characteristics and long-term follow-up. Int J Epidemiol. 2011;40(6):1652–66. Epub 2011/12/14. 10.1093/ije/dyr120 22158673 PMC3235021

[13] Bennett D, Li L, Du H, Guo Y, Bian Z, Chen J, et al Physical activity and the incidence of major cardiovascular diseases: Evidence from the China Kadoorie Biobank Study. Eur Heart J. 2015;36:465.

[14] Sadarangani KP, Hamer M, Mindell JS, Coombs NA, Stamatakis E. Physical activity and risk of all-cause and cardiovascular disease mortality in diabetic adults from Great Britain: pooled analysis of 10 population-based cohorts. Diabetes Care. 2014;37(4):1016–23. Epub 2014/03/22. 10.2337/dc13-1816 24652727

[15] Du H, Bennett D, Li L, Whitlock G, Guo Y, Collins R, et al Physical activity and sedentary leisure time and their associations with BMI, waist circumference, and percentage body fat in 0.5 million adults: the China Kadoorie Biobank study. Am J Clin Nutr. 2013;97(3):487–96. Epub 2013/02/01. 10.3945/ajcn.112.046854 23364014 PMC4345799

[16] Clarke R, Shipley M, Lewington S, Youngman L, Collins R, Marmot M, et al Underestimation of risk associations due to regression dilution in long-term follow-up of prospective studies. Am J Epidemiol. 1999;150(4):341–53. Epub 1999/08/24. 10453810 10.1093/oxfordjournals.aje.a010013

[17] Lampe JW. Health effects of vegetables and fruit: assessing mechanisms of action in human experimental studies. Am J Clin Nutr. 1999;70(3 Suppl):475S–90S. Epub 1999/09/09. 10479220 10.1093/ajcn/70.3.475s

[18] Nakamura K, Nagata C, Oba S, Takatsuka N, Shimizu H. Fruit and vegetable intake and mortality from cardiovascular disease are inversely associated in Japanese women but not in men. J Nutr. 2008;138(6):1129–34. Epub 2008/05/22. 18492845 10.1093/jn/138.6.1129

[19] MacMahon S, Peto R, Cutler J, Collins R, Sorlie P, Neaton J, et al Blood pressure, stroke, and coronary heart disease. Part 1, Prolonged differences in blood pressure: prospective observational studies corrected for the regression dilution bias. Lancet. 1990;335(8692):765–74. Epub 1990/03/31. 1969518 10.1016/0140-6736(90)90878-9

[20] Frost C, Thompson SG. Correcting for regression dilution bias: comparison of methods for a single predictor variable. Journal of the Royal Statistical Society Series A (Statistics in Society). 2000;163(2):173–89.

[21] Armstrong ME, Green J, Reeves GK, Beral V, Cairns BJ. Frequent physical activity may not reduce vascular disease risk as much as moderate activity: large prospective study of women in the United Kingdom. Circulation. 2015;131(8):721–9. Epub 2015/02/18. 10.1161/CIRCULATIONAHA.114.010296 25688148

[22] Sofi F, Capalbo A, Cesari F, Abbate R, Gensini GF. Physical activity during leisure time and primary prevention of coronary heart disease: an updated meta-analysis of cohort studies. Eur J Cardiovasc Prev Rehabil. 2008;15(3):247–57. Epub 2008/06/06. 10.1097/HJR.0b013e3282f232ac 18525378

[23] Wang X, Ouyang Y, Liu J, Zhu M, Zhao G, Bao W, et al Fruit and vegetable consumption and mortality from all causes, cardiovascular disease, and cancer: systematic review and dose-response meta-analysis of prospective cohort studies. BMJ. 2014;349:g4490 Epub 2014/07/31. 10.1136/bmj.g4490 25073782 PMC4115152

[24] Woodcock J, Franco OH, Orsini N, Roberts I. Non-vigorous physical activity and all-cause mortality: systematic review and meta-analysis of cohort studies. Int J Epidemiol. 2011;40(1):121–38. Epub 2010/07/16. 10.1093/ije/dyq104 20630992

[25] Barzi F, Woodward M, Marfisi RM, Tavazzi L, Valagussa F, Marchioli R. Mediterranean diet and all-causes mortality after myocardial infarction: results from the GISSI-Prevenzione trial. Eur J Clin Nutr. 2003;57(4):604–11. Epub 2003/04/18. 10.1038/sj.ejcn.1601575 12700623

[26] de Lorgeril M, Salen P, Martin JL, Monjaud I, Delaye J, Mamelle N. Mediterranean diet, traditional risk factors, and the rate of cardiovascular complications after myocardial infarction: final report of the Lyon Diet Heart Study. Circulation. 1999;99(6):779–85. Epub 1999/02/17. 9989963 10.1161/01.cir.99.6.779

[27] Li S, Flint A, Pai JK, Forman JP, Hu FB, Willett WC, et al Dietary fiber intake and mortality among survivors of myocardial infarction: prospective cohort study. BMJ. 2014;348:g2659 Epub 2014/05/02. 10.1136/bmj.g2659 24782515 PMC4004785

[28] Trichopoulou A, Bamia C, Norat T, Overvad K, Schmidt EB, Tjonneland A, et al Modified Mediterranean diet and survival after myocardial infarction: the EPIC-Elderly study. Eur J Epidemiol. 2007;22(12):871–81. Epub 2007/10/11. 10.1007/s10654-007-9190-6 17926134

[29] Larsson SC, Virtamo J, Wolk A. Total and specific fruit and vegetable consumption and risk of stroke: a prospective study. Atherosclerosis. 2013;227(1):147–52. Epub 2013/01/09. 10.1016/j.atherosclerosis.2012.12.022 23294925

[30] Du H, Li L, Bennett D, Guo Y, Turnbull I, Yang L, et al Fresh fruit consumption in relation to incident diabetes and diabetic vascular complications: a 7-year prospective study of 0.5 million Chinese adults. PLoS Medicine. 2017; 14(4):e1002279 10.1371/journal.pmed.1002279 28399126 PMC5388466

[31] Du H, Li L, Whitlock G, Bennett D, Guo Y, Bian Z, et al Patterns and socio-demographic correlates of domain-specific physical activities and their associations with adiposity in the China Kadoorie Biobank study. BMC Public Health. 2014;14:826 Epub 2014/08/12. 10.1186/1471-2458-14-826 25106853 PMC4138397

[32] Smith SC Jr, Benjamin EJ, Bonow RO, Braun LT, Creager MA, Franklin BA, et al AHA/ACCF Secondary Prevention and Risk Reduction Therapy for Patients with Coronary and other Atherosclerotic Vascular Disease: 2011 update: a guideline from the American Heart Association and American College of Cardiology Foundation. Circulation. 2011;124(22):2458–73. Epub 2011/11/05. 10.1161/CIR.0b013e318235eb4d 22052934

[33] Thompson PD, Buchner D, Pina IL, Balady GJ, Williams MA, Marcus BH, et al Exercise and physical activity in the prevention and treatment of atherosclerotic cardiovascular disease: a statement from the Council on Clinical Cardiology (Subcommittee on Exercise, Rehabilitation, and Prevention) and the Council on Nutrition, Physical Activity, and Metabolism (Subcommittee on Physical Activity). Circulation. 2003;107(24):3109–16. Epub 2003/06/25. 10.1161/01.CIR.0000075572.40158.77 12821592

[34] Pate RR, Pratt M, Blair SN, Haskell WL, Macera CA, Bouchard C, et al Physical activity and public health. A recommendation from the Centers for Disease Control and Prevention and the American College of Sports Medicine. JAMA. 1995;273(5):402–7. Epub 1995/02/01. 7823386 10.1001/jama.273.5.402

[35] Myers J, Prakash M, Froelicher V, Do D, Partington S, Atwood JE. Exercise capacity and mortality among men referred for exercise testing. N Engl J Med. 2002;346(11):793–801. Epub 2002/03/15. 10.1056/NEJMoa011858 11893790

[36] Billinger SA, Arena R, Bernhardt J, Eng JJ, Franklin BA, Johnson CM, et al Physical activity and exercise recommendations for stroke survivors: a statement for healthcare professionals from the American Heart Association/American Stroke Association. Stroke. 2014;45(8):2532–53. Epub 2014/05/23. 10.1161/STR.0000000000000022 24846875

[37] Leenders M, Sluijs I, Ros MM, Boshuizen HC, Siersema PD, Ferrari P, et al Fruit and vegetable consumption and mortality: European prospective investigation into cancer and nutrition. Am J Epidemiol. 2013;178(4):590–602. Epub 2013/04/20. 10.1093/aje/kwt006 23599238

[38] Oyebode O, Gordon-Dseagu V, Walker A, Mindell JS. Fruit and vegetable consumption and all-cause, cancer and CVD mortality: analysis of Health Survey for England data. J Epidemiol Community Health. 2014;68(9):856–62. Epub 2014/04/02. 10.1136/jech-2013-203500 24687909 PMC4145465

[39] Ning M, Zhang Q, Yang M. Comparison of self-reported and biomedical data on hypertension and diabetes: findings from the China Health and Retirement Longitudinal Study (CHARLS). BMJ Open. 2016;6(1):e009836 Epub 2016/01/06. 10.1136/bmjopen-2015-009836 26729390 PMC4716227

[40] Okura Y, Urban LH, Mahoney DW, Jacobsen SJ, Rodeheffer RJ. Agreement between self-report questionnaires and medical record data was substantial for diabetes, hypertension, myocardial infarction and stroke but not for heart failure. J Clin Epidemiol. 2004;57(10):1096–103. Epub 2004/11/06. 10.1016/j.jclinepi.2004.04.005 15528061

[41] Yusuf S, Islam S, Chow CK, Rangarajan S, Dagenais G, Diaz R, et al Use of secondary prevention drugs for cardiovascular disease in the community in high-income, middle-income, and low-income countries (the PURE Study): a prospective epidemiological survey. Lancet. 2011;378(9798):1231–43. Epub 2011/08/30. 10.1016/S0140-6736(11)61215-4 21872920

[42] Kernan WN, Ovbiagele B, Black HR, Bravata DM, Chimowitz MI, Ezekowitz MD, et al Guidelines for the prevention of stroke in patients with stroke and transient ischemic attack: a guideline for healthcare professionals from the American Heart Association/American Stroke Association. Stroke. 2014;45(7):2160–236. Epub 2014/05/03. 10.1161/STR.0000000000000024 24788967

[43] Teo K, Lear S, Islam S, Mony P, Dehghan M, Li W, et al Prevalence of a healthy lifestyle among individuals with cardiovascular disease in high-, middle- and low-income countries: The Prospective Urban Rural Epidemiology (PURE) study. JAMA. 2013;309(15):1613–21. Epub 2013/04/18. 10.1001/jama.2013.3519 23592106

