# Supplementary material for: Fruit consumption and physical activity in relation to all-cause and cardiovascular mortality among 70,000 Chinese adults with pre-existing vascular disease
Source: PLoS One. 2017 Apr 12;12(4):e0173054. doi: 10.1371/journal.pone.0173054 (PMC5389797; doi:10.1371/journal.pone.0173054)
Supplement: S1 Fig — Analyses were stratified by age-at-risk, sex, region, and baseline CVD status, and adjusted for education, income, smoking, consumption of alcohol, dairy products, meat and preserved vegetables, survey season, diabetes status, family history of CVD, CVD medication, poor health status, and physical activity, where appropriate. The black boxes represent HRs and the horizontal bars represent their confidence intervals. The open diamonds represent the overall estimates of HRs and their confidence intervals. 1. the HR after correcting for regression dilution bias; 2. the HR before correcting for regression dilution bias. (DOCX) [file pone.0173054.s001.docx]

**S1 Fig. Adjusted HRs of 1 daily portion of fresh fruit consumption associated with all-cause and CVD mortality by subgroups of participants.**

| 1. **All-cause mortality**   **** | 1. **CVD mortality**   **** |
| --- | --- |
