# Supplementary material for: Fruit consumption and physical activity in relation to all-cause and cardiovascular mortality among 70,000 Chinese adults with pre-existing vascular disease
Source: PLoS One. 2017 Apr 12;12(4):e0173054. doi: 10.1371/journal.pone.0173054 (PMC5389797; doi:10.1371/journal.pone.0173054)
Supplement: S1 Table — *The mean daily portion number came from the 2nd resurvey data, used as a proxy of baseline mean daily portion. †Usual intake amount for each group was estimated by taking into account changes in consumption frequency between baseline and 1st resurvey using this formula Un=∑i=13(Fni×Bi); F is the percentage in each cell, B is the baseline proportion per month for each baseline category, U is the usual proportion per month for each baseline category. This method for correcting for regression dilution bias is equivalent to the MacMahon-Peto method, described in detail by Frost C and Thompson SG. In Correcting for regression dilution bias: comparison of methods for a single predictor variable.[Journal of the Royal Statistical Society Series A (Statistics in Society) 2000, 163(2):173–189]. (DOCX) [file pone.0173054.s003.docx]

**eTable1. Calculation of usual fruit consumption using data from the 1^st^ and 2^nd^ resurvey (n = 2690)**

| Baseline | | 1^st^ resurvey (mean 2.6 years later) | | | Consumption frequency (d/mth) | Mean daily portion* | Baseline consumption (portions/month) | Usual consumption (portions/month)† |
| --- | --- | --- | --- | --- | --- | --- | --- | --- |
|  |  | < 1 days/wk  1 | - 1. days/wk   2 | >3 days/wk  3 |  |  |  |  |
| 1 | < 1 day/wk | 63.40%  (589) | 24.87%  (231) | 11.73%  (109) | 1.5 | 0.81 | B_1_ = 1.2 (1.5×0.81) | 8.30 |
| 2 | 1-3 days/wk | 35.53%  (297) | 35.17%  (294) | 29.31%  (245) | 8.6 | 1.43 | B_4_ = 12.3 (8.6×1.43) | 15.95 |
| 3 | >3 days/wk | 9.84%  (91) | 20.86%  (193) | 69.30%  (641) | 23.6 | 1.62 | B_5_ = 38.2 (23.6×1.62) | 29.16 |
|  |  |  |  |  |  |  |  |  |
|  | Difference (3-1) |  |  |  |  |  | 37.0 | 20.86 |
|  | MacMahon-Peto regression dilution ratio | | | |  |  | 0.56 | |

*The mean daily portion number came from the 2nd resurvey data, used as a proxy of baseline mean daily portion.

†Usual intake amount for each group was estimated by taking into account changes in consumption frequency between baseline and 1st resurvey using this formula Un = $\sum_{i=1}^{3} (Fni\times Bi)$; F is the percentage in each cell, B is the baseline proportion per month for each baseline category, U is the usual proportion per month for each baseline category. This method for correcting for regression dilution bias is equivalent to the MacMahon-Peto method, described in detail by Frost C and Thompson SG. In ***Correcting for regression dilution bias: comparison of methods for a single predictor variable***.[Journal of the Royal Statistical Society Series A (Statistics in Society) 2000, 163(2):173-189]
