# Supplementary material for: Fruit consumption and physical activity in relation to all-cause and cardiovascular mortality among 70,000 Chinese adults with pre-existing vascular disease
Source: PLoS One. 2017 Apr 12;12(4):e0173054. doi: 10.1371/journal.pone.0173054 (PMC5389797; doi:10.1371/journal.pone.0173054)
Supplement: S2 Table — Values are either percentage or mean (SD) and were adjusted for age, sex, and study area where appropriate. * Stroke group included all participants with self-reported physician-diagnosed stroke, among which 1142 also had IHD; IHD group included those with self-reported IHD, but not stroke; Hypertension group included participants with self-reported hypertension, but without stroke or IHD. † In men, the proportion of current smokers was 47.9% and the proportion of current drinkers was 27.0%; the corresponding proportions in women were 2.5% and 1.5% respectively. ‡ Regular consumption means consuming food products for at least 4 days per week. ¶ Overweight was defined as BMI≥24 kg/m2 and uncontrolled hypertension was defined as SBP≥140 mmHg or DBP≥90 mmHg or both. § Includes aspirin, statins, calcium antagonist, beta-receptor blockers, ice-inhibitors, diuretics or other unspecified drugs. ¥ Either self-rated poor health or reported having a low capacity of walk. (DOCX) [file pone.0173054.s004.docx]

| **eTable 2. Baseline characteristics of participants by baseline prevalent disease*** | | | | |
| --- | --- | --- | --- | --- |
|  | **Stroke or TIA**  (n=8171) | **IHD**  (n=13,936) | **Hypertension**  (n=47,940) | **Overall**  (n = 70,047) |
| **Age (SD), years** | 61.4 (9.2) | 61.1 (9.3) | 57.8 (9.2) | 58.9 (9.3) |
| **Women, %** | 46.1 | 64.1 | 61.7 | 60.3 |
| **Urban population, %** | 59.6 | 65.8 | 50.1 | 54.4 |
| **High school or above, %** | 40.0 | 46.4 | 43.6 | 43.8 |
| **Annual income>20,000 Yuan, %** | 41.8 | 47.2 | 46.9 | 46.4 |
| **Smoking, %** |  |  |  |  |
| Never regular | 66.8 | 67.7 | 69.9 | 69.1 |
| Former regular | 12.8 | 11.8 | 9.5 | 10.4 |
| Current regular | 20.4 | 20.5 | 20.6 | 20.5^†^ |
| **Alcohol consumption, %** |  |  |  |  |
| Never regular | 84.1 | 85.5 | 83.8 | 84.1 |
| Former regular | 8.4 | 4.8 | 3.4 | 4.3 |
| Current regular | 7.5 | 9.7 | 12.8 | 11.6^†^ |
| **Regular food consumption**^‡^**, %** |  |  |  |  |
| Fresh fruit | 29.2 | 34.9 | 33.0 | 32.9 |
| Fresh vegetables | 98.6 | 98.8 | 98.5 | 98.6 |
| Preserved vegetables | 21.8 | 23.3 | 23.7 | 23.4 |
| Meat | 42.7 | 46.7 | 48.0 | 47.1 |
| Dairy products | 27.0 | 31.8 | 26.1 | 27.4 |
| **Physical activity (SD), MET-hr/day** | 13.6 (10.3) | 15.4 (10.4) | 16.4 (10.2) | 15.8 (11.8) |
| **BMI (SD), kg/m^2^** | 24.6 (3.5) | 24.8 (3.5) | 25.3 (3.4) | 25.1 (3.5) |
| **Overweight**^¶^**, %** | 55.8 | 57.9 | 64.2 | 62.0 |
| **SBP (SD), mmHg** | 144.9 (22.9) | 137.8 (22.9) | 153.1 (22.6) | 149.1 (23.3) |
| **DBP (SD), mmHg** | 82.8 (11.8) | 79.3 (11.9) | 85.9 (11.7) | 84.2 (12.4) |
| **Uncontrolled hypertension**^¶^**, %** | 56.9 | 43.3 | 73.9 | 65.8 |
| **Family history of CVD, %** | 32.8 | 28.4 | 28.7 | 29.1 |
| **Diabetes, %** | 15.9 | 14.0 | 14.2 | 14.4 |
| **CVD medication**^§^**,%** | 71.5 | 65.8 | 78.1 | 74.9 |
| **Self-reported poor health**^¥^**, %** | 41.4 | 37.7 | 19.6 | 25.8 |

Values are either percentage or mean (SD) and were adjusted for age, sex, and study area where appropriate.

* Stroke group included all participants with self-reported physician-diagnosed stroke, among which 1142 also had IHD; IHD group included those with self-reported IHD, but not stroke; Hypertension group included participants with self-reported hypertension, but without stroke or IHD.

† In men, the proportion of current smokers was 47.9% and the proportion of current drinkers was 27.0%; the corresponding proportions in women were 2.5% and 1.5% respectively.

‡ Regular consumption means consuming food products for at least 4 days per week.

¶ Overweight was defined as BMI≥24 kg/m^2^ and uncontrolled hypertension was defined as SBP≥140 mmHg or DBP≥90 mmHg or both.

§ Includes aspirin, statins, calcium antagonist, beta-receptor blockers, ice-inhibitors, diuretics or other unspecified drugs.

¥ Either self-rated poor health or reported having a low capacity of walk.
