# Supplementary material for: Fruit consumption and physical activity in relation to all-cause and cardiovascular mortality among 70,000 Chinese adults with pre-existing vascular disease
Source: PLoS One. 2017 Apr 12;12(4):e0173054. doi: 10.1371/journal.pone.0173054 (PMC5389797; doi:10.1371/journal.pone.0173054)
Supplement: S3 Table — (DOCX) [file pone.0173054.s005.docx]

**eTable 3: Distribution of total deaths at 35-79 years during follow-up**

| **Causes of death** | **ICD-10 codes** | **With baseline CVD (n = 22,107)** | | **Without CVD (n = 47,940)** | | **Total (n=70,047)** | |
| --- | --- | --- | --- | --- | --- | --- | --- |
|  |  | **No. of deaths** | **Proportion (%)** | **No. of deaths** | **Proportion (%)** | **No. of deaths** | **Proportion (%)** |
| **Total vascular** | I00-I25, I28-I99 | 1761 | 61.0 | 1804 | 49.0 | 3565 | 54.3 |
| Total CVD | I00-I25, I28-I88, I95-I99 | 1760 | 61.0 | 1803 | 49.0 | 3563 | 54.2 |
|  |  |  |  |  |  |  |  |
| **Total cancer** | C00-C97 | 578 | 20.0 | 988 | 26.8 | 1566 | 23.8 |
|  |  |  |  |  |  |  |  |
| **Total respiratory diseases** | J00-J99, I26-I27 | 210 | 7.3 | 277 | 7.5 | 487 | 7.4 |
|  |  |  |  |  |  |  |  |
| **Other known causes** | D00-H95, K00-Q99, S00-T98 | 192 | 6.7 | 308 | 8.4 | 500 | 7.6 |
|  |  |  |  |  |  |  |  |
| **Infectious & parasitic disease, ill-defined causes, external causes, or missing** | A00-B99, R00-R99, V01-Y98 | 145 | 5.0 | 306 | 8.3 | 451 | 6.9 |
|  |  |  |  |  |  |  |  |
| **All-cause death** |  | 2886 | 100 | 3683 | 100 | 6569 | 100 |
