# Supplementary material for: Fruit consumption and physical activity in relation to all-cause and cardiovascular mortality among 70,000 Chinese adults with pre-existing vascular disease
Source: PLoS One. 2017 Apr 12;12(4):e0173054. doi: 10.1371/journal.pone.0173054 (PMC5389797; doi:10.1371/journal.pone.0173054)
Supplement: S4 Table — IHD: Ischemic heart disease; TIA: transient ischaemic attack. *1142 participants also had IHD Analyses were stratified by age-at-risk, sex, region, and baseline CVD status, and adjusted for education, income, smoking, consumption of alcohol, dairy products, meat and preserved vegetables, survey season, diabetes status, family history of CVD, CVD medication, poor health status, and fruit consumption or physical activity, where appropriate. (DOCX) [file pone.0173054.s006.docx]

**eTable 4. Separate associations of fresh fruit consumption and physical activity with all-cause and CVD mortality in people with prevalent stroke and those with IHD at baseline.**

| Mortality | Associations with fruit consumption | | | | |
| --- | --- | --- | --- | --- | --- |
|  | <1 day/week | 1-3 days/week | >3 days/week | | Per 100 g/day |
|  | Among people with prevalent stroke or TIA at baseline (n=8171)* | | | | |
| Overall |  |  |  | |  |
| No. of deaths | 675 | 387 | 382 | | 1444 |
| HR (95%CI) | 1.0 (0.90-1.11) | 0.89 (0.81-0.98) | 0.91 (0.81-1.03) | | 0.88 (0.69-1.13) |
| CVD |  |  |  | |  |
| No. of deaths | 480 | 261 | 227 | | 968 |
| HR (95%CI) | 1.0 (0.88-1.13) | 0.90 (0.81-1.01) | 0.86 (0.73-0.99) | | 0.79 (0.58-1.08) |
|  | Among people with only IHD at baseline (n=13,936) | | | | |
| Overall |  |  | |  |  |
| No. of deaths | 587 | 407 | | 448 | 1442 |
| HR (95%CI) | 1.0 (0.90-1.11) | 1.03 (0.94-1.13) | | 0.93 (0.83-1.04) | 0.88 (0.71-1.10) |
| CVD |  |  | |  |  |
| No. of deaths | 335 | 233 | | 224 | 956 |
| HR (95%CI) | 1.0 (0.87-1.15) | 1.09 (0.97-1.23) | | 0.92 (0.78-1.08) | 0.86 (0.64-1.17) |
| Mortality | Associations with physical activity | | | | |
|  | 1^st^ tertile | 2^nd^ tertile | | 3^rd^ tertile | Per 10 MET-hr/day |
|  | Among people with prevalent stroke or TIA at baseline (n=8171)* | | | | |
| Overall |  |  | |  |  |
| No. of deaths | 939 | 332 | | 173 | 1444 |
| HR (95%CI) | 1.0 (0.93-1.07) | 0.77 (0.69-0.86) | | 0.61 (0.51-0.72) | 0.65 (0.56-0.74) |
| CVD |  |  | |  |  |
| No. of deaths | 645 | 218 | | 105 | 968 |
| HR (95%CI) | 1.0 (0.92-1.09) | 0.74 (0.64-0.85) | | 0.51 (0.41-0.63) | 0.54 (0.45-0.65) |
|  | Among people with only IHD at baseline (n=13,936) | | | | |
| Overall |  |  | |  |  |
| No. of deaths | 790 | 454 | | 198 | 1442 |
| HR (95%CI) | 1.0 (0.93-1.08) | 0.80 (0.73-0.88) | | 0.59 (0.50-0.70) | 0.62 (0.54-0.71) |
| CVD |  |  | |  |  |
| No. of deaths | 436 | 241 | | 115 | 792 |
| HR (95%CI) | 1.0 (0.90-1.11) | 0.78 (0.68-0.88) | | 0.66 (0.53-0.81) | 0.66 (0.55-0.80) |

IHD: Ischemic heart disease; TIA: transient ischaemic attack

*1142 participants also had IHD
