# Supplementary material for: Fruit consumption and physical activity in relation to all-cause and cardiovascular mortality among 70,000 Chinese adults with pre-existing vascular disease
Source: PLoS One. 2017 Apr 12;12(4):e0173054. doi: 10.1371/journal.pone.0173054 (PMC5389797; doi:10.1371/journal.pone.0173054)
Supplement: S5 Table — Analyses were stratified by age-at-risk, sex, region, and baseline CVD status, and adjusted for education, income, smoking, consumption of alcohol, dairy products, meat and preserved vegetables, survey season, diabetes status, family history of CVD, CVD medication, poor health status, and fruit consumption or physical activity, where appropriate. (DOCX) [file pone.0173054.s007.docx]

**eTable 5. Results from sensitivity analyses.**

| Mortality | **Associations with fruit consumption** | | | | |
| --- | --- | --- | --- | --- | --- |
|  | <1 day/week | 1-3 days/week | >3 days/week | | Per 100 g/day |
|  | **Exclude first 2 years follow-up** | | | | |
| Overall |  |  |  | |  |
| No. of deaths | 2441 | 1537 | 1304 | | 5282 |
| HR (95%CI) | 1.0 (0.95-1.05) | 0.92 (0.88-0.97) | 0.83 (0.78-0.88) | | 0.76 (0.68-0.86) |
| CVD |  |  |  | |  |
| No. of deaths | 1426 | 816 | 640 | | 2882 |
| HR (95%CI) | 1.0 (0.93-1.07) | 0.92 (0.86-0.98) | 0.80 (0.73-0.88) | | 0.72 (0.61-0.85) |
|  | **Exclude first 2 years follow-up and those with poor health status at baseline** | | | | |
| Overall |  |  | |  |  |
| No. of deaths | 1391 | 978 | | 883 | 3252 |
| HR (95%CI) | 1.0 (0.94-1.07) | 0.91 (0.86-0.96) | | 0.85 (0.78-0.92) | 0.79 (0.68-0.92) |
| CVD |  |  | |  |  |
| No. of deaths | 789 | 487 | | 410 | 1686 |
| HR (95%CI) | 1.0 (0.91-1.10) | 0.88 (0.81-0.96) | | 0.81 (0.72-0.91) | 0.74 (0.60-0.92) |
|  | **Additionally adjusted for consumption of fresh vegetables** | | | | |
| Overall |  |  | |  |  |
| No. of deaths | 3094 | 1880 | | 1595 | 6569 |
| HR (95% CI) | 1.0 (0.96-1.05) | 0.91 (0.87-0.95) | | 0.84 (0.79-0.89) | 0.78 (0.70-0.86) |
| CVD |  |  | |  |  |
| No. of deaths | 1797 | 996 | | 770 | 3563 |
| HR (95% CI) | 1.0(0.94-1.06) | 0.91 (0.86-0.96) | | 0.79(0.73-0.86) | 0.72 (0.62-0.83) |
|  | **Additionally adjusted for consumption of whole-grain staple foods** | | | | |
| Overall |  |  | |  |  |
| No. of deaths | 3094 | 1880 | | 1595 | 6569 |
| HR (95% CI) | 1.0 (0.96-1.05) | 0.91 (0.87-0.95) | | 0.84 (0.79-0.89) | 0.77 (0.70-0.86) |
| CVD |  |  | |  |  |
| No. of deaths | 1797 | 996 | | 770 | 3563 |
| HR (95% CI) | 1.0 (0.94-1.06) | 0.90 (0.85-0.96) | | 0.79 (0.72-0.86) | 0.71 (0.61-0.83) |
|  | **Exclude 180 participants with baseline prevalent cancer** | | | | |
| Overall |  |  | |  |  |
| No. of deaths | 3069 | 1849 | | 1557 | 6475 |
| HR (95% CI) | 1.0 (0.96-1.05) | 0.90 (0.86-0.94) | | 0.83 (0.78-0.88) | 0.77 (0.69-0.85) |
| CVD |  |  | |  |  |
| No. of deaths | 1794 | 988 | | 760 | 3542 |
| HR (95% CI) | 1.0 (0.94-1.06) | 0.90 (0.85-0.95) | | 0.78 (0.72-0.85) | 0.71 (0.61-0.82) |
|  | **Exclude 10,074 participants with baseline prevalent diabetes** | | | | |
| Overall |  |  | |  |  |
| No. of deaths | 2455 | 1504 | | 1245 | 5204 |
| HR (95% CI) | 1.0 (0.95-1.05) | 0.90 (0.86-0.94) | | 0.85 (0.79-0.91) | 0.79 (0.69-0.89) |
| CVD |  |  | |  |  |
| No. of deaths | 1455 | 806 | | 602 | 2863 |
| HR (95% CI) | 1.0 (0.93-1.07) | 0.90 (0.85-0.96) | | 0.82 (0.74-0.90) | 0.75 (0.63-0.89) |
|  | **Include 109,682 extra participants with baseline blood pressure > 140/90 mmHg** | | | | |
| Overall |  |  | |  |  |
| No. of deaths | 7311 | 3872 | | 2686 | 13,869 |
| HR (95% CI) | 1.0 (0.97-1.03) | 0.92 (0.90-0.95) | | 0.82 (0.79-0.86) | 0.74 (0.68-0.80) |
| CVD |  |  | |  |  |
| No. of deaths | 3643 | 1774 | | 1190 | 6607 |
| HR (95% CI) | 1.0 (0.96-1.04) | 0.91 (0.88-0.95) | | 0.78 (0.73-0.83) | 0.68 (0.60-0.77) |
| Mortality | **Associations with physical activity** | | | | |
|  | 1^st^ tertile | 2^nd^ tertile | | 3^rd^ tertile | Per 10 MET-hr/day |
|  | **Exclude first 2 years follow-up** | | | | |
| Overall |  |  | |  |  |
| No. of deaths | 2620 | 1523 | | 1139 | 5282 |
| HR (95%CI) | 1.0 (0.96-1.04) | 0.79 (0.75-0.83) | | 0.68 (0.63-0.73) | 0.76 (0.71-0.80) |
| CVD |  |  | |  |  |
| No. of deaths | 1501 | 811 | | 570 | 2882 |
| HR (95%CI) | 1.0 (0.94-1.06) | 0.76 (0.71-0.81) | | 0.64 (0.58-0.70) | 0.69 (0.63-0.75) |
|  | **Exclude first 2 years follow-up and those with poor health status at baseline** | | | | |
| Overall |  |  | |  |  |
| No. of deaths | 1475 | 1002 | | 775 | 3252 |
| HR (95%CI) | 1.0 (0.94-1.06) | 0.83 (0.78-0.88) | | 0.69 (0.63-0.75) | 0.76 (0.71-0.82) |
| CVD |  |  | |  |  |
| No. of deaths | 802 | 515 | | 369 | 1686 |
| HR (95%CI) | 1.0 (0.92-1.08) | 0.81 (0.74-0.88) | | 0.63 (0.56-0.72) | 0.67 (0.59-0.75) |
|  | **Additionally adjusted for consumption of fresh vegetables** | | | | |
| Overall |  |  | |  |  |
| No. of deaths | 3282 | 1864 | | 1423 | 6569 |
| HR (95% CI) | 1.0 (0.96-1.04) | 0.78 (0.75-0.82) | | 0.66 (0.62-071) | 0.74 (0.70-0.78) |
| CVD |  |  | |  |  |
| No. of deaths | 1859 | 978 | | 726 | 3563 |
| HR (95% CI) | 1.0 (0.95-1.05) | 0.74 (0.70-0.79) | | 0.63 (0.58-0.69) | 0.69 (0.64-0.74) |
|  | **Additionally adjusted for consumption of whole-grain staple foods** | | | | |
| Overall |  |  | |  |  |
| No. of deaths | 3282 | 1864 | | 1423 | 6569 |
| HR (95% CI) | 1.0 (0.96-1.04) | 0.78 (0.75-0.82) | | 0.67 (0.63-0.71) | 0.74 (0.70-0.79) |
| CVD |  |  | |  |  |
| No. of deaths | 1859 | 978 | | 726 | 3563 |
| HR (95% CI) | 1.0 (0.95-1.05) | 0.74 (0.70-0.79) | | 0.64 (0.58-0.69) | 0.69 (0.64-0.74) |
|  | **Excluded 180 participants with baseline prevalent cancer** | | | | |
| Overall |  |  | |  |  |
| No. of deaths | 3233 | 1831 | | 1411 | 6475 |
| HR (95% CI) | 1.0 (0.96-1.04) | 0.78 (0.74-0.81) | | 0.67 (0.63-0.71) | 0.75 (0.71-0.79) |
| CVD |  |  | |  |  |
| No. of deaths | 1850 | 967 | | 725 | 3542 |
| HR (95% CI) | 1.0 (0.95-1.05) | 0.74 (0.69-0.78) | | 0.63 (0.58-0.69) | 0.69 (0.64-0.74) |
|  | **Exclude 10,074 participants with baseline prevalent diabetes** | | | | |
| Overall |  |  | |  |  |
| No. of deaths | 2509 | 1463 | | 1232 | 5204 |
| HR (95% CI) | 1.0 (0.96-1.04) | 0.79 (0.75-0.83) | | 0.67 (0.63-0.72) | 0.76 (0.71-0.80) |
| CVD |  |  | |  |  |
| No. of deaths | 1454 | 779 | | 630 | 2863 |
| HR (95% CI) | 1.0 (0.94-1.06) | 0.74 (0.69-0.79) | | 0.62 (0.57-0.68) | 0.69 (0.63-0.75) |
|  | **Include 109,682 extra participants with baseline blood pressure > 140/90 mmHg** | | | | |
| Overall |  |  | |  |  |
| No. of deaths | 6590 | 4134 | | 3145 | 13,869 |
| HR (95% CI) | 1.0 (0.97-1.03) | 0.78 (0.76-0.81) | | 0.67 (0.64-0.70) | 0.87 (0.86-0.89) |
| CVD |  |  | |  |  |
| No. of deaths | 3388 | 1865 | | 1354 | 6607 |
| HR (95% CI) | 1.0 (0.96-1.04) | 0.73 (0.70-0.76) | | 0.65 (0.61-0.69) | 0.85 (0.83-0.88) |
