# Supplementary material for: Fruit consumption and physical activity in relation to all-cause and cardiovascular mortality among 70,000 Chinese adults with pre-existing vascular disease
Source: PLoS One. 2017 Apr 12;12(4):e0173054. doi: 10.1371/journal.pone.0173054 (PMC5389797; doi:10.1371/journal.pone.0173054)
Supplement: S6 Table — Analyses were stratified by age-at-risk, sex, region, and baseline CVD status, and adjusted for education, income, smoking, consumption of alcohol, dairy products, meat and preserved vegetables, survey season, diabetes status, family history of CVD, CVD medication, poor health status, and fruit consumption or physical activity, where appropriate. (DOCX) [file pone.0173054.s008.docx]

**eTable 6. Fresh fruit consumption and physical activity in relation to non-fatal CVD events**

| Mortality | Association with fruit consumption | | | |
| --- | --- | --- | --- | --- |
|  | <1 day/week | 1-3 days/week | >3 days/week | 1 daily portion |
| Total major adverse cardiovascular events | | |  |  |
| No. of deaths | 4494 | 3369 | 3826 | 11,689 |
| HR (95% CI) | 1.0 (0.96-1.04) | 0.99 (0.96-1.02) | 0.90 (0.87-0.94) | 0.86 (0.80-0.93) |
| IHD |  |  |  |  |
| No. of deaths | 3337 | 2901 | 4162 | 10,400 |
| HR (95% CI) | 1.0 (0.96-1.04) | 0.99 (0.96-1.03) | 0.95 (0.92-0.99) | 0.93 (0.85-1.01) |
| Total stroke | |  |  |  |
| No. of deaths | 4242 | 3209 | 3658 | 11,109 |
| HR (95% CI) | 1.0 (0.96-1.04) | 0.99 (0.95-1.02) | 0.89 (0.86-0.93) | 0.84 (0.78-0.92) |
| Ischemic stroke | |  |  |  |
| No. of deaths | 3574 | 2819 | 3395 | 9788 |
| HR (95% CI) | 1.0 (0.96-1.04) | 1.02 (0.98-1.05) | 0.93 (0.89-0.97) | 0.89 (0.82-0.97) |
| Intracerebral haemorrhage | |  |  |  |
| No. of deaths | 800 | 474 | 370 | 1644 |
| HR (95% CI) | 1.0 (0.91-1.09) | 0.86 (0.79-0.93) | 0.79 (0.70-0.89) | 0.71 (0.57-0.89) |
| Mortality | Association with physical activity | | | |
|  | 1^st^ tertile | 2^nd^ tertile | 3^rd^ tertile | 10 MET-hr/day |
| Total major adverse cardiovascular events | | |  |  |
| No. of deaths | 5154 | 3963 | 2572 | 11,689 |
| HR (95% CI) | 1.0 (0.97-1.03) | 0.91 (0.88-0.94) | 0.82 (0.78-0.85) | 0.86 (0.82-0.89) |
| IHD |  |  |  |  |
| No. of deaths | 4419 | 3787 | 2194 | 10,400 |
| HR (95% CI) | 1.0 (0.97-1.03) | 0.96 (0.93-0.99) | 0.88 (0.84-0.92) | 0.90 (0.86-0.95) |
| Total stroke | | |  |  |
| No. of deaths | 4864 | 3790 | 2455 | 11,109 |
| HR (95% CI) | 1.0 (0.97-1.03) | 0.92 (0.89-0.95) | 0.82 (0.78-0.86) | 0.86 (0.82-0.90) |
| Ischemic stroke | | |  |  |
| No. of deaths | 4379 | 3375 | 2034 | 9788 |
| HR (95% CI) | 1.0 (0.97-1.03) | 0.92 (0.89-0.95) | 0.82 (0.78-0.86) | 0.85 (0.81-0.89) |
| Intracerebral haemorrhage | | |  |  |
| No. of deaths | 656 | 519 | 469 | 1644 |
| HR (95% CI) | 1.0 (0.92-1.09) | 0.90 (0.83-0.98) | 0.79 (0.71-0.88) | 0.88 (0.80-0.97) |
